# Retirement due to disabilities in patients with type 1 diabetes a nationwide multicenter survey in Brazil

**DOI:** 10.1186/s12889-015-1812-4

**Published:** 2015-05-12

**Authors:** Marilia Brito Gomes, Carlos Antonio Negrato, On behalf of the Brazilian Type 1 Diabetes Study Group (BrazDiab1SG)

**Affiliations:** Department of Internal Medicine, Diabetes Unit, State University of Rio de Janeiro, Rio de Janeiro, Brazil; Bauru’s Diabetics Association, Bauru, São Paulo Brazil

**Keywords:** Type 1 diabetes, Chronic complications, Early retirement, Disabilities

## Abstract

**Background:**

There is scarcity of data concerning retirement and workforce loss exclusively in patients with type 1 diabetes. The aim of this study was to evaluate the prevalence, causes, and predictors of retirement in patients with type 1 diabetes in Brazil.

**Methods:**

This was a multicenter cross-sectional study conducted between December 2008 and December 2010 in 28 public clinics in 20 Brazilian cities. Data were obtained from 3,180 patients aged 22 ± 11.7 years; 56.3% of the participants were female, and 57.4% were Caucasians. The mean time since diabetes diagnosis was 10.3 ± 8.1 years. Patients’ information (clinical factors and retirement data) was obtained through a questionnaire and a chart review. Patients were retired by diabetes according to the Brazilian Institute of Social Security’s criteria that takes in account the presence of diabetes-related chronic complications certified by a doctor, excluding any personal reason or another health condition besides diabetes*.* Both quantitative and qualitative tests were employed, and a multivariate logistic regression model was performed to identify the factors associated with retirement due to disabilities in patients with type 1 diabetes.

**Results:**

The overall frequency of retirement was 4.2%, with no difference between genders. The mean age of retirement was 35.5 ± 9.3 years, resulting in 17.5 ± 9.1 years of workforce losses. These patients had a significantly higher prevalence of severe hypoglycemia, proliferative and non-proliferative retinopathy, foot disorders, chronic or end-stage renal disease requiring dialysis or transplantation, cataracts, glaucoma, psychological disorders, hypertension, and overweight/obesity than did the employed patients. Multivariate logistic regression analysis with retirement as the dependent variable showed adjusted odds ratios (ORs) of 4.87 (2.66-8.78) for the presence of microvascular complications and 3.7 (2.04-6.70) for macrovascular complications.

**Conclusions:**

Retirement due to disabilities occurred in 4.2% of Brazilian patients with type 1 diabetes at an early age and is strongly associated with diabetes-related chronic complications. Health care workers should thus reevaluate the quality of care given to these patients.

## Background

The incidence of type 1 diabetes (T1D) is increasing worldwide along with its chronic complications and has become a major health problem and financial burden because of its high direct and indirect costs [1-7]. The indirect costs of diabetes are primarily related to productivity losses due to disability, early retirement, unemployment, and absenteeism caused by acute and chronic complications [4,8,9]. Patients with diabetes generally participate less in the workforce than subjects without diabetes of the same age and gender, as indicated by the indirect costs of diabetes reported by the Health and Retirement Study (HRS) conducted in the US [10]. At baseline, patients with diabetes in the HRS were less educated and had more comorbidities, such as hypertension, coronary artery disease, stroke, congestive heart failure, visual impairment, kidney and foot problems, than individuals without diabetes [10]. This study as well as the GAZEL study conducted in France [11] have linked diabetes with greater risks of disability, retirement, and early death. However, these studies were conducted in predominantly middle-aged patients with type 2 diabetes. Another study conducted in the US evaluating the sociodemographic characteristics of persons with diabetes has found that patients with T1D have a lower probability of employment and higher rates of absenteeism and early retirement [12]. However, it is important to emphasize that so far there are few data in the international literature including Brazil on this topic, which is a very important issue because of its long-lasting consequences for patients and society.

This study aimed to evaluate the prevalence, and clinical factors related to retirement in patients with T1D in Brazil.

## Methods

This multicenter, retrospective, cross-sectional, observational study was conducted between December 2008 and December 2010 in 28 public secondary (ambulatory outpatient clinics) and tertiary care-level clinics (ambulatory outpatient clinics in university hospitals) located in 20 cities in four Brazilian geographic regions (north/northeast, mid-west, southeast, and south). The details of the data collection methods have been published previously [13,14]. All patients received healthcare from the Brazilian National Health Care System (BNHCS). All eligible participating centers had a diabetes clinic with at least one endocrinologist which provided data from a minimum of 50 consecutive outpatients with diagnosis of T1D who regularly attended the clinic. The inclusion criteria consisted of a diagnosis of T1D by a physician that was based on the typical clinical presentation, including variable degrees of weight loss, polyuria, polydipsia and polyphagia, and the need for continuous insulin use since the diagnosis of T1D. All patients were diagnosed between 1960 and 2010. Patients who did not fulfill these criteria were excluded from the study. A total of 3,591 patients were enrolled.

The ethics committee form the Pedro Ernesto University Hospital that belongs to the State University of Rio de Janeiro approved the study as did all the local ethics committees of each center (Appendix 1). Written informed consent was obtained from all patients or their parents, as appropriate.

Demographic, educational, economic, and working status data and the following variables were assessed in an interview during a clinical visit: age, age at diagnosis, duration of diabetes, blood pressure, modalities of treatment, comorbidities, frequent severe hypoglycemia (self-reported), and smoking status. The levels of glycated hemoglobin (HbA1c), fasting plasma glucose (FPG), total cholesterol, low-density lipoprotein (LDL) cholesterol, high-density lipoprotein (HDL) cholesterol, and triglycerides measured at the last clinical visit were obtained from medical records. The screenings for retinopathy,(by fundoscopy; classified as absent, non-proliferative, or proliferative [15]), clinical nephropathy (by microalbuminuria, classified according to ADA recommendations [15]), macrovascular diseases (clinical coronary artery disease, stroke, and peripheral vascular disease), and foot pathologies in patients with diabetes duration equal or greater than five years were evaluated in medical records when these procedures were performed within one year of the study assessment. Hospitalization for diabetes ketoacidosis, hyperglycemia without DKA and hypoglycemia in the previous year were also investigated.

The following ADA goals for adequate metabolic and clinical control [15] were adopted by the BrazDiab1SG: HbA1c < 7%; systolic blood pressure (sBP) < 130 mmHg; diastolic blood pressure (dBP) < 80 mmHg; body mass index (BMI) < 25 kg/m^2^; FPG < 130 mg/dl (7.2 mmol/l); total cholesterol < 200 mg/dl (5.2 mmol/l); HDL cholesterol > 40 mg/dl for men (1.1 mmol/l) and > 50 mg/dl (1.3 mmol/l) for women; LDL cholesterol < 100 mg/dl (2.6 mmol/l); non-HDL cholesterol < 130 mg/dl (3.30 mmol/l) and triglycerides < 150 mg/dl/l (1.7 mmol/l).

Hypertension was defined as sBP ≥ 140 mmHg and/or dBP ≥ 90 mmHg, as measured during the last clinical visit [8] or self-reported. Overweight was defined as a BMI ≥ 25 kg/m^2^, and obesity was defined as a BMI ≥ 30 kg/m^2^ [15]. The HbA1c values obtained in the last clinical visit in 2,768 patients (87%) were measured using a method certified by the National Glycohemoglobin Standardization Program (NGSP), namely, high-performance liquid chromatography (1,514 patients, 54.7%) or turbidimetry (1,254 patients, 45.3%). HbA1c levels that were determined using other methods, missing data, and HbA1c measurements obtained more than one year prior to the study assessment were excluded from the glycemic control analyses (n = 412, 13%). FPG, triglycerides, HDL cholesterol, and total cholesterol were measured using enzymatic techniques. LDL cholesterol was calculated using Friedewald’s equation [16]. Current smoking was defined as smoking more than one cigarette per day at the time of the interview.

### Sample calculation, economic status

A detailed description of the study sample calculation has been described previously [13,14]. Briefly, the study sample represented the distribution of T1D cases across four geographic regions of Brazil. This distribution was estimated using the overall population distribution reported in the 2000 Brazilian Institute of Geography and Statistics Population Census (IBGE) [17]. These data were combined with national estimates of diabetes prevalence derived from a 1988 survey to determine the minimum number of patients to be studied in each region [18]. Economic status was defined according to the Brazilian Economic Classification Criteria [19]. This classification also accounts for education level: illiterate/incomplete primary education, complete primary education/incomplete secondary education, complete secondary education/incomplete high school, complete high school/some college, or college graduate. The following economic status categories were considered for this analysis: high, middle, low, and very low [19].

### Sample of the present study

**Figure 1:**
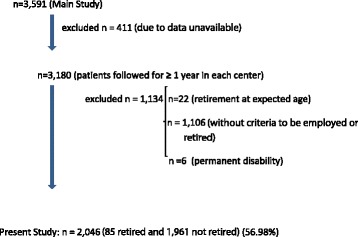
Flow chart of the patients included in the present study.

In the present study we included patients with at least one year of medical follow-up at each respective center (n = 3,180) and patients who fulfilled the criteria of Brazilian Institute of Social Security (BISS), which determines that only individuals older than 15 years could be employed, and only individuals with at least one year of continuous employment could be retired [20]. We also excluded from the present study patients with T1D who retired at the expected age (n = 22, 0.7%), patients who presented no criteria to be either employed or retired (n = 1,106, 34.8%), and patients with permanent disability due to other diseases [n = 6, 0.2% (Down syndrome, n = 4; congenital amaurosis, n = 1; and schizophrenia, n = 1)] . The final sample of the present study was n = 2,046 patients which is summarized in the flow chart described in Figure 1. Patients were retired by diabetes according to the Brazilian Institute of Social Security’s criteria that takes in account the presence of diabetes-related chronic complications certified by a doctor, excluding any personal reason or another health condition besides diabetes [20]. Workforce loss was estimated based on the age at retirement because of T1D and the age of retirement in the general Brazilian population, which is 55 years for men and 52 years for women [20]. After this age, workers quit their labor activity and start receiving benefits from the BISS. An individual is considered to be early retired when receiving benefits for any reason, including by disabilities due to diabetes, before the above mentioned age [20].

### Statistical analysis

The data were presented as the means (± SD) or median (minimum-maximum) for continuous variables and as counts (relative frequencies) for discrete variables. All the variables were tested for normality distribution. When a normal distribution was observed parametric tests were used; otherwise non-parametric tests were applied. A multivariate logistic regression model was performed with retirement (yes/no) as the dependent (outcome) variable and the presence of microvascular and macrovascular complications as the independent (exposure) variables. Other predictive variables, such as socioeconomic status, ethnicity (Caucasian or non-Caucasian, based on self-reporting), age, geographic region, gender, and duration of diabetes, were controlled in the analysis using a stepwise regression analysis. The following steps were applied for modeling: (1) baseline model (social and demographic characteristics, such as gender, ethnicity, social class and region), (2) introduction of the variable disease duration and (3) final models, with introduction of the independent variables: minor complications and major complications. Workforce losses were only estimated based on age of retirement of Brazilian men (55 years) and women (52 years). All analyses were performed using the Statistical Package for the Social Sciences (SPSS version 17.0, SPSS, Inc., Chicago, Illinois, USA). Odds ratios with 95% confidence intervals (CIs) were calculated when indicated. A two-sided p value less than 0.05 was considered significant.

## Results

### Overview of participant demographics, socioeconomic status, level of care, and insulin treatment

Data were obtained from 3,180 patients (excluded n = 411, 11.5%). The average follow-up time in each respective diabetes center was 6.1 (1-49) years. The economic status of 2,154 (67.8%) of the patients was either very low [n=1,102 (34.7%)] or low [n=1,052 (33.1%)]*.* Combined intermediate or long-acting insulin and short-acting insulin was the most common therapeutic regimen [2,334 (84.3%) patients]. Most patients reported using three or more daily injections of short-acting insulin (1,785 patients, 56.2%) and three or more SBGM daily tests (1,907 patients, 60%). The demographic data of this population are listed in Table 1.

**Table 1:** **Clinical and demographic data of the source population**

**Variable**	
**N**	3,180
**Female**, **n** (%)	1,791 (56.3)
**Age**, **y**	22 ± 11.8
**Age range**, **y**, **n** (%)	
0-4.9	51 (1.6)
5-9.9	308 (9.7)
10-14.9	604 (19)
15-29.9	1,471 (46.3)
30 or older	746 (23.5)
**Ethnicity**, **n** (%)	
Caucasian	1,824 (57.4)
Non-Caucasian*	1,356 (42.6)
Economic status**	
High	222 (7.2)
Medium	710 (22.3)
Low	1,052 (33.1)
Very low	1,102 (34.7)
**Retirement**, **n** (%)	
No criteria for retirement***	1,106 (34.8)
Retired at the expected age	22 (0.7)
Not retired	1,961 (61.7)
Retired because of DM	85 (2.7)
**Level of care n** (%)	
Secondary	897 (28.2)
Tertiary	2,283 (71.8)
**Duration of diabetes**, **y**	10.3 ± 8.04

y = year; data are presented as number (percentage) or mean ± SD.

*African-Brazilians, Mulattos, Asians, and Native Indians.

**Missing data from 87 participants; ***Patients younger than 16 y.

### Overview of the overall frequency of retirement, demographic data, level of care and related workforce losses

The overall frequency of retirement due to diabetes in patients who met the BISS criteria of retirement was 4.2% with no difference between men and women. No difference in the mean age of retirement due to diabetes was observed between genders (men: 37.4 ± 9.4 vs women: 34.4 ± 9.0, p = 0.14). The mean duration of retirement due to diabetes was 6 (<1-30) years. The time of follow-up in each center was greater in patients who had retired because of diabetes than in patients who were employed (12.3 ± 9.8 years vs 7.9 ± 6.1 years, p < 0.001). The proportion of patients who were treated at the tertiary care level center was higher in the retired group than in the employed group [n = 74 (87.1%) vs 1,414 (72.1%), p = 0.001]

The estimated mean workforce losses in years in patients who had retired due to diabetes was almost 17 years in both genders. The demographic, clinical, and laboratory data of patients who retired because of T1D and employed patients are compared in Table 2.

**Table 2:** **Economic and medical variables in retired type 1 diabetes patients and employed patients**

**Variable**	Retirement*		***P*** *-* **value**
	**Yes (%)**	**No (%)**	
**N**	85 (4.2)	1,961 (95.8)	-
**Age**	42.7 ± 10.6	26.9 ± 9.2	<0.001
**Age at diagnosis of diabetes,** ***y***	18.5 ± 9.1	14.2 ± 7.9	<0.001
**Female**, **n (%)**	44 (51.8)	1,120 (57.1)	0.27
**Duration of diabetes,** ***y***	24.2 ± 9.1	12.7 ± 7.7	<0.001
Ethnicity*			0.05
Caucasian	57 (67.4)	1,120 (57.1)	
Non-Caucasian*	28 (32.6)	841 (42.9)	
**Economic status,** **n** **(%)**			0.01
High	2 (2.3)	195 (9.9)	
Medium	21 (25.6)	529 (26.9)	
Low	27 (31.1)	690 (35.2)	
Very low	35 (40.7)	547 (27.9)	
**Geographical region**			0.02
North/Northeast	13 (15.1)	589 (30.1)	
Southeast	44 (51.2)	776 (39.6)	
South	23 (27.9)	472 (24.1)	
Midwest	5 (5.8)	123 (6.3	
**BMI**	24.6 ± 4.0	23.5 ± 3.6	0.005
**Overweight or obesity, n (%)**	36 (42.9)	615 (31.5)	0.03
**Fasting glycemia** ***(mg/dL)***	191.2 ± 115.5	177.7 ± 104.1	0.2
**HbA1c (%)HbA1c (mmol/mol)**	8.9 ± 1.874 ± 19.7	9.3 ± 2.378 ± 25.1	0.2
**HbA1c <7.0%, n (%)**	6 (7.6)	244 (14.4)	0.9
**HbA1c ≥9.0%, n (%)**	34 (43.0)	799 (47.2)	0.2
**sBP (mmHg)**	128.1 ± 16.7	116.8 ± 16.0	<0.001
**dBP (mmHg)**	79.8 ± 9.8	74.9 ± 10.8	<0.001
**Total cholesterol** ***(mg/dL)***	173.9 ± 44.2	173.2 ± 43.3	0.8
**Triglycerides** ***(mg/dL)***	121.8 ± 116.6	95.3 ± 70.8	0.001
**HDL cholesterol** ***(mg/dL)***	54.6 ± 18.3	53.1 ± 15.2	0.4
**LDL cholesterol** ***(mg/dL)***	96.4 ± 34.6	101.5 ± 34.5	0.19
**Current smoker, y (%)**	4 (4.7)	123 (6.3)	0.06
**Insulin dose (U/kg/day)**	0.68 ± 0.35	0.87 ± 0.35	<0.001
**Number of clinical visits (previous year)**	4.3 ± 1.7	3.9 ± 1.6	0.01
**Chronic complications, n (%)**			
Microvascular	68 (80.0)	538 (31.6)	<0.0001
Macrovascular	27 (31.8)	85 (5.0)	<0.0001
Micro and macrovascular	69 (81.2)	575 (33.8)	<0.0001
**Foot pathologies, n (%)**			
Ulcer	4 (4.7)	31 (1.8)	0.16
Amputation	9 (10.6)	9 (0.5)	<0.0001
Charcot arthropathy	2 (2.4)	5 (0.3)	0.01
**Severe hypoglycemia**	22 (37.9)	228 (18.5)	0.007

Overweight and obesity were considered together.

The data are presented as number (percentage) or mean ± SD.

*African-Brazilian, Mulatto, Asian, or Native Indian.

### Overview of frequency of retirement due to diabetes and the coexistence of cardiovascular risk factors

Overweight or obesity was noted in 651 patients (31.8%). Patients who retired due to diabetes were more likely to be overweight or obese than were employed patients [36 (42.9%) vs 615 (31.5%), p <0.03]. Hypertension was observed in 563 (27.5%) patients. The frequency of hypertension was greater among those who had retired because of diabetes than among employed patients [61 (72.6%) *vs* 502 (26.1%), p <0.0001]. There were no between-group differences in the proportions of patients who achieved their target LDL cholesterol, total cholesterol or HDL cholesterol levels. Fewer patients who had retired because of diabetes reached their triglycerides targets [62 (76.5%) *vs* 1432 (84.2%), p <0.07]. Overall, 486 (23.8%) patients were receiving antihypertensive agents, and 253 (12.4%) patients were receiving statins. Patients who had retired because of diabetes were more likely to use antihypertensive agents than employed patients [48 (56.5%) *vs* 438 (22.3%), p < 0.0001)] and were also more likely to use statins [28 (32.5%) *vs* 225 (11.5%), p < 0.0001].

### Overview of diabetes treatment modality, glycemic control, and the coexistence of acute and chronic diabetes-related complications in patients with retirement

Patients who had retired because of diabetes were less likely to use combined insulin therapy than were employed patients [58 (69.9%) vs 1,638 (84.8%), p = 0.0002]. No differences in the total number of insulin injections, total number of daily SBGM tests, or HbA1c levels were observed between patients who had retired because of diabetes and employed patients.

No differences in the frequencies of hospitalization because of hypoglycemia, hyperglycemia or diabetes ketoacidosis were observed between patients who had retired because of diabetes and employed patients [8 (9.4%) vs 145 (7.4%), p = 0.6] and [2 (2.4%) vs 85 (4.3%), p = 0.4].

Considering the sample of the present study (n = 2,046), among patients who had undergone fundoscopy in the previous year (n = 1,596; 78%), patients who had retired because of diabetes were more likely than were employed patients to have proliferative retinopathy or non-proliferative retinopathy [32 (43.2%) vs 122 (8.7%), p < 0.0001 and 23 (31.1%) vs 156 (11.2%), p < 0.0001, respectively]. More patients who had retired because of diabetes than employed patients had undergone laser therapy and vitrectomy [43 (50.6%) vs 137 (8.1%), p < 0.0001 and 7 (8.2%) vs 27 (1.6%), p < 0.0001, respectively]. Additionally, higher proportions of the retired patients than of the employed patients had cataracts and glaucoma [13 (15.3%) *vs* 100 (5.9%), p < 0.0001 and 7 (7.1%) *vs* 58 (3.4%), p < 0.001, respectively].

Furthermore, among patients for whom renal function data had been measured within the previous year (n = 1,529;75%), those who had retired because of diabetes were more likely to have clinical nephropathy [11 (12.9%) vs 84 (4.9%)], chronic renal disease receiving conservative treatment [6 (7.1%) vs 36 (2.1%)], or chronic renal disease requiring dialysis treatment [7 (8.2%) vs 16 (1.0%)] or to have undergone renal transplantation [5 (5.9%) vs 4 (0.2%)] (p < 0.010 for all comparisons). No difference was observed concerning demographic and clinical data between patients who had or not information about the status of retinopathy or nephropathy (data not shown).

Other complications, such as cataracts (n = 2), severe hypoglycemia (n = 2), and psychological disorders (n = 5) without any chronic complications, were observed only in patients with retirement because of diabetes. The distribution of diabetes-related complications is shown in Table 2.

Using unadjusted multivariate analysis with retirement because of diabetes as the dependent variable, the odds ratios (ORs) given the presence of macrovascular and microvascular complications were 9.02 (95% CI 5.43-14.97, p < 0.001) and 9.20 (95% CI 5.29-16.02, p < 0.001), respectively. The adjusted models are shown in Tables 3 and 4. The discriminatory capacity and adjustment parameters of the models is showed in Table 5*.*

**Table 3:** **Final adjusted logistic regression model of early retirement because of type 1 diabetes and the presence of microvascular complications in Brazil (n = 2,046)**

	**N**	**OR (95% CI)**	**Adjusted** ***P-*** **value**
**Microvascular complications**			
Yes	606	4.87 (2.71-8.78)	<0.001
No	1,181	-	Reference
**Socioeconomic status** ^**a**^			
High	197	-	Reference
Medium	550	6.57 (1.31-32.97)	0.022
Low	717	6.81 (1.37-33.80)	0.019
Very low	582	10.58 (2.15-52.16)	0.004
**Age** ^**b**^	2,046	1.09 (1.06-1.12)	<0.001
**Duration of diabetes** ^**b**^	2,046	1.05 (1.02-1.09)	0.004

^a^Reference: high socioeconomic status.

^b^Continuous variables.

**Table 4:** **Final adjusted logistic regression model of early retirement because of type 1 diabetes and the presence of macrovascular complications in Brazil (n = 2,046)**

	**N**	**OR (95% CI)**	**Adjusted** ***P-*** **value**
**Macrovascular complications**			
Yes	112	3.70 (2.04-6.70)	<0.001
No	1,675	-	Reference
**Socioeconomic status** ^**a**^			
High	197		
Medium	550	9.08 (1.69-48.9)	0.010
Low	717	8.67 (1.63-46.05)	0.011
Very low	582	13.42 (2.54-70.92)	0.002
**Age** ^**b**^	2,046	1.08 (1.05-1.11)	<0.001
**Duration of diabetes** ^**b**^	2,046	1.06 (1.03-1.10)	<0.001

^a^Reference: high socioeconomic status.

^b^Continuous variables.

**Table 5:** **Discriminatory capacity and adjustment of models for predicting retirement**

**Model**	**-2 log likelihood**	**Cox & Snell R Square**	**Nagelkerke R Square**
Baseline model^a^	521,934	,087	,296
Baseline model + duration of disease^b^	508,358	,093	,317
Final model: Model 2 + microvascular complication	468,837	,110	,349
Final model: Model 2 + macrovascular complication	485,017	,102	,324

^a^Social and demographic characteristics: gender, age, ethnicity, region, and socioeconomic status.

^b^Gender, ethnicity, and region were excluded due to lack of statistical significance.

## Discussion

To the best of our knowledge, this is the first study addressing retirement exclusively in patients with T1D.

We have found that in our sample of patients with T1D who met the criteria of BISS for retirement, 4.2% were already retired. The majority of these patients with retirement because of diabetes did it early, and had either acute (frequent severe hypoglycemia) or chronic diabetes-related complications and were from low or very low socioeconomic status. Our results provide evidence of a significant impact of T1D and related complications causing disabilities and early retirement in Brazil. We have found that among our young cohort, the majority were on working age and in the most productive years of their lives.

The association of disabilities caused by diabetes with early retirement is largely diabetes-related chronic complications, such as retinopathy, nephropathy, macrovascular disease, and foot pathologies, and similar complications were observed in both genders. Our data are similar to those of the HRS conducted in the US [10], which showed that retired individuals with diabetes generally had more comorbidities and a higher probability of self-reported disability than those without diabetes.

Other complications, such as cataracts, glaucoma, severe hypoglycemia, and psychological disorders without any chronic complications, were observed only in patients with early retirement due to disabilities caused by diabetes. According to a study conducted in Spain, which did not evaluate data concerning early retirement, severe hypoglycemia had significant direct and indirect impacts on healthcare costs [8].

Patients that retired early used less complex insulin treatment regimens, were more frequently treated at secondary care clinics, and were more likely to be of medium, low or very low socioeconomic status compared with those patients who were not retired. Although the BNHCS is universal, and every Brazilian citizen can look for free medical attention, when people retire their earnings become lower than when they are actively working. This will generally imply in a worsening of their socioeconomic status.

Our study has also shown that glycemic control was not associated with early retirement due to disabilities caused by diabetes. However, this finding must be interpreted with caution because more than 40% of our patients had HbA1c levels greater than 9.0% and because it is difficult to establish causal relationships in cross-sectional studies such as ours. The Diabetes Control and Complications Trial (DCCT) [21] and DCCT/Epidemiology of Diabetes Interventions and Complications Study (EDIC) [22] have shown that effective diabetes control decreases the risk of developing chronic microvascular complications [21,22]. Recently, the EDIC showed that excess weight gain in DCCT was associated with the presence of metabolic syndrome markers [23]. Our patients who retired early due to disabilities caused by diabetes had more comorbidities, such as hypertension, overweight/obesity and increased triglycerides levels.

Considering that intensive management of glycemia, weight, cholesterol, and blood pressure are effective in delaying or preventing diabetes-related complications [24-27], it is imperative to change the approach for these patients to a proactive model that could probably prevent the occurrence of diabetes-related chronic complications and thus the probability of early retirement due to disabilities.

The mean age of retirement due to disabilities caused by diabetes in our patients was 34.0 years for women and 37.4 years for men. This is a worrying situation considering that the standard age of retirement in the general population in our country is 52 years for women and 55 years for men [20]. These data show that early retirement in patients due to disabilities caused by diabetes results in more than 15 years of workforce loss per retired patient in Brazil.

Retirement generally coincides with deterioration of one's health and correlates with increasing age; these statistics may predict greater expenses for the public health systems in both developing and developed countries [28]. In Brazil, each patient with T1D represents an average annual direct medical cost of 1319.15 US dollars [29]. We have no national data about the amount of annual indirect costs spent with this setting of patients and this must be addressed in future studies.

The strengths of our study include its large sample size that represents the diverse, young Brazilian population with T1D and includes a wide range of ethnic groups and socioeconomic backgrounds from all geographic regions of the country. The data were collected with a uniform, standardized recruitment protocol which provided data from a minimum of 50 consecutive outpatients with diagnosis of T1D who regularly attended the clinics in all participating centers and therefore represent a large occupational cohort.

However, our study has several limitations that must be addressed. One limitation was the sample characteristics and study design. All patients lived in large cities and received care from a specialist at a public health center; thus, patients who relied on primary care facilities and lived in rural areas may not have been considered. However, the latter group represents a small minority of T1D patients receiving treatment in Brazil (less than 1% according to the diabetes prevalence survey conducted in 1988) [18]. Our study design was retrospective, cross-sectional, mainly exploratory so it did not allow us to establish a causal relationship. Another limitation of our study was the absence of psychosocial evaluation. Family support and patient self-efficacy have been associated with positive outcomes including better glycemic control, which in many cases could help to avoid or postpone T1D-related complications and consequent early retirement due to disabilities. The occurrence of self-reported hypoglycemia as the cause of retirement in two patients without diabetes-related chronic complications could also be considered a limitation of our study. Other limitation was related to the relationship between the evaluation of clinical outcomes and retirement, since the majority of the retired patients had retired before this evaluation.

## Conclusion

In conclusion, 4.2% of our patients who met the BISS criteria to be retired had early retirement mainly because of diabetes-related chronic complications. Thus, it is imperative that Brazilian diabetes care teams change the education and follow-up approaches used for patients with T1D. A more proactive model would help prevent or postpone the occurrence of chronic complications and consequently early retirement due to disabilities caused by diabetes, which may in many ways worsen an already compromised quality of life. Further studies are necessary to establish the costs of productivity losses due to early retirement due to disabilities caused by diabetes. Finally, early retirement due to disabilities must be considered an important issue in the subset of patients with T1D.

## Abbreviations

T1D: Type 1 diabetes
ADA: American Diabetes Association
HRS: Health and Retirement Study
GAZEL: The GAZ and Électricité cohort study
NHIS: National Health Interview Survey
BrazDiab1SG: Brazilian Type 1 Diabetes Study Group
BNHCS: Brazilian National Health Care System
BISS: Brazilian Institute of Social Security
HbA_1_C: glycated hemoglobin
NGSP: National Glycohemoglobin Standardization Program
FBG: Fasting blood glucose
BDS: Brazilian Diabetes Society
sBP: Systolic blood pressure
dBP: Diastolic blood pressure
BMI: Body mass index
HDL: High-density lipoprotein
LDL: Low-density lipoprotein
SBGM: Self-blood glucose monitoring

## References

[1] Karvonen M, Vik-Kajander M, Moltchanova E, Libman I, LaPorte R, Tuomilehto J (2000). Incidence of childhood type 1 diabetes worldwide. Diabetes Care.

[2] The DIAMOND Project Group (2006). Incidence and trends of childhood type 1 diabetes worldwide 1990–1999. Diabet Med.

[3] Negrato CA, Dias JP, Teixeira MF, Dias A, Salgado MH, Lauris JR (2010). Temporal trends in incidence of type 1 diabetes between 1986 and 2006 in Brazil. J Endocrinol Invest.

[4] Laing SP, Swerdlow AJ, Slater SD, Burden AC, Morris A, Waugh NR (2003). Mortality from heart disease in a cohort of 23,000 patients with insulin-treated diabetes. Diabetologia.

[5] Miller RG, Secrest AM, Sharma RK, Songer TJ, Orchard TJ (2012). Improvements in the life expectancy of type 1 diabetes: The Pittsburgh epidemiology of diabetes complications study cohort. Diabetes.

[6] Wild S, Roglic G, Green A, Sicree R, King H (2004). Global prevalence of diabetes, Estimates for the year 2000 and projections for 2030. Diabetes Care.

[7] American Diabetes Association (2007). Economic costs of diabetes in the US in 2007. Diabetes Care.

[8] Brito-Sanfiel M, Diago-Cabezudo J, Calderon A (2010). Economic impact of hypoglycemia on healthcare in Spain. Expert Rev of Pharmacoecon Outcomes Res.

[9] Ng YC, Jacobs P, Johnson JA (2001). Productivity losses associated with diabetes in the U.S. Diabetes Care.

[10] Vijan S, Hayward RA, Langa KM (2004). The impact of diabetes on workforce participation: results from a national household sample. Health Serv Res.

[11] Herquelot E, Guéguen A, Bonenfant S, Dray-Spira R (2011). . Impact of diabetes on work cessation. Data from the GAZEL cohort study. Diabetes Care.

[12] CC Cowie, MS Eberhardt. Sociodemographic characteristics of persons with diabetes. In Diabetes in America 2^nd^ ed. Harris M, Cowie C, Stern M, Boyko E, Reiber G, Bennet P, Eds. Bethsed, MD, National Institute of Health 1995, 85-116.

[13] Gomes MB, Coral M, Cobas RA, Dib SA, Canani LH, Nery M (2012). On behalf of the Brazilian type 1 diabetes study group (BrazDiab1SG). prevalence of adults with type 1 diabetes who meet the goals of care in daily clinical practice: a nationwide multicenter study in brazil. Diabetes Res Clin Prac..

[14] Gomes MB, de Mattos Matheus AS, Calliari LE, Luescher JL, Manna TD, Savoldelli RD (2013). On behalf of the Brazilian type 1 diabetes study group (BrazDiab1SG). economic status and clinical care in young type 1 diabetes patients: a nationwide multicenter study in brazil. Acta Diabetol.

[15] American Diabetes Association (2013). Clinical practice recommendations. Diabetes Care.

[16] Friedwald WT, Levy R, Fredrickson DS (1972). Estimations of serum low density lipoprotein cholesterol without use of preparative ultracentrifuge. Clin Chem.

[17] Instituto Brasileiro de Geografia e Estatística (IBGE). Censo 2000 [acessed in 2008 aug]. Available from http:/www.ibge.gov.br/censo.

[18] Malerbi DA, Franco LJ (1992). Multicenter study of the prevalence of diabetes mellitus and impaired glucose tolerance in the urban Brazilian population aged 30-69 yr. The Brazilian Cooperative Group on the Study of Diabetes Prevalence Diabetes Care.

[19] ABEP. Brazilian Economic classification criteria, 2010 [accessed in 2008 aug]. Available from http://www.abep.org.

[20] Instituto Seguridade Social. [accessed in 2013, February]. Available from http://www.previdenciasocial.gov.br/.

[21] DCCT (1993). The Diabetes Control and Complications Trial. N. Engl. J. Med.

[22] The Diabetes Control and Complications Trial/Epidemiology of Diabetes Interventions and Complications (DCCT/EDIC) Study Research Group. Intensive diabetes treatment and cardiovascular disease in patients with diabetes type 1. N. Engl. J. Med. 2005, 353: 2643-53. 10.1056/NEJMoa052187 PMC2637991 16371630

[23] Purnell JQ, Zinman B, Brunzell JD (2013). And for the DCCT/EDIC research group.The effect of excess weight gain with intensive diabetes mellitus treatment on cardiovascular risk factors and atherosclerosis in type 1 diabetes mellitus: results from the diabetes control and complications trial/epidemiology of diabetes interventions and complications (DCCT/EDIC) study. Circulation.

[24] EeG-Olofsson K, Cederrholm J, Nilsson PM, Gudbjornsdóttir S, Eliasson B (2007). For the steering committee of the swedish national diabetes register. Glycemic and risk factor control in type 1 diabetes. Diabetes Care.

[25] Zgibor JC, Wilson RR, Orchard TJ (2005). Has control of hypercholesterolemia and hypertension in type 1 diabetes improver over time?. Diabetes Care.

[26] Orchard TJ, Forrest KY, Kuller LH, Becker DJ (2001). Lipid and blood pressure treatment goals for type 1 diabetes 10- year incidence data from the Pittsburgh epidemiology of diabetes complications study. Diabetes Care.

[27] Moraes CM, Portela RB, Pinheiro VS (2003). Prevalência de sobrepeso e obesidade em diabéticos tipo 1. Arq Bra Endocrinol Metab.

[28] Gould CE, Beaudreau SA (2013). Association between depression and anxiety on blood pressure dysregulation and pulse in the health and retirement study. Int J Geriatr Psychiatry.

[29] Cobas RA, Ferraz MB, Matheus AS, Tannus LR, Negrato CA, de Araujo A (2013). Brazilian type 1 diabetes study group
. The cost of type 1 diabetes: a nationwide multicentre study in brazil. Bull World Health Organ.

